# Supplementary material for: Phylogeny and Mycotoxin Characterization of Alternaria Species Isolated from Wheat Grown in Tuscany, Italy
Source: Toxins (Basel). 2018 Nov 14;10(11):472. doi: 10.3390/toxins10110472 (PMC6267338; doi:10.3390/toxins10110472)
Supplement: Supplementary file 1 [file toxins-10-00472-s001.pdf]

Supplementary Materials: Phylogeny and Mycotoxin Characterization of Alternaria Species Isolated from Wheat Grown in Tuscany, Italy

Francesca A. Ramires, Mario Masiello, Stefania Somma, Alessandra Villani, Antonia Susca, Antonio F. Logrieco, Carlos Luz, Giuseppe Meca and Antonio Moretti

Table S1. Mean value and range of fungal contamination detected in the 100 wheat samples collected throughout the Tuscany region over four consecutive crop seasons (2013–2016).

Host	Year	Number of Fields	Fungal Contamination (%)											
			Alternaria spp.		*Fusarium* spp.		*Cladosporium* spp.		*Epicoccum* spp.		*Stemphylium* spp.		Other Fungal Genera	
			Range	Mean Value	Range	Mean Value	Range	Mean Value	Range	Mean Value	Range	Mean Value	Range	Mean Value
**Soft Wheat**	2013	10	0–31	17	0–1	0.3	12–26	19	1–26	16	10–21	15	10–81	24
	2014	16	0–17	4	0–9	2	6–33	18	3–31	16	6–22	16	4–34	21
	2015	11	4–73	26	0–3	1	6–31	17	5–21	16	1–21	11	3–33	13
	2016	11	11–50	33	0–7	3	9–33	16	10–24	16	5–15	10	0–72	21
**Durum Wheat**	2013	31	0–48	17	0–14	3	0–22	13	0–22	14	0–18	11	0–33	15
	2014	15	0–11	7	0–2	0.3	11–35	23	10–26	17	10–37	24	2–25	13
	2015	4	8–43	26	0–6	3	10–29	16	10–29	22	10–20	16	2–15	8
	2016	2	22–35	29	3–13	6	10–32	21	9–15	11	4–26	18	4–13	9

Table S2. Phylogenetic clades reported in Figure 1 of the 134 strains identified using multilocus sequence approach, isolated from 100 wheat samples collected throughout the Tuscany region over four consecutive crop seasons (2013–2016).

Years of Sampling	Host	Strains	Clade				
			A1	A2	A5	D	E
2013	Soft wheat	13	17,876; 17,880; 17,881; 17,890; 17,903	17,867	17,902	17,877; 17,879; 17,884; 17,887; 17,891	17,904
	Durum wheat	43	17,862; 17,874; 17,875; 17,882; 17,886; 17,889; 17,892; 17,894; 17,895; 17,896; 17,897; 17,898; 17,899; 17,901; 17,905, 17,907; 17,908; 17,909; 17,910; 17,912	17,857, 17,860, 17,883	17,858; 17,865; 17,868; 17,872; 17,873; 17,878; 17,885; 17,888; 17,893; 17,900; 17,906; 17,911	17,859; 17,861, 17,863; 17,864, 17,866; 17,869, 17,870; 17,871	
2014	Soft wheat	16	17,913; 17,915; 17,929; 17,930; 17,932; 17,933; 17,934; 17,938; 17,940; 17,942		17,914; 17,939	17,916; 17,935	17,931; 17,936
	Durum wheat	16	17,917; 17,918; 17,920; 17,923; 17,924; 17,926; 17,928; 17,937;17,943; 17,944	17,921, 17,922, 17,927	17,919; 17,925	17,941	
2015	Soft wheat	20	17,945; 17,950; 17,952; 17,953; 17,954; 17,956; 17,958; 17,961; 17,962; 17,963; 17,964; 17,965; 17,967	17,959, 17,969		17,966; 17,955, 17,968; 17,957, 17,946	
	Durum wheat	8	17,960; 17,972	17,971	17,947; 17,948; 17,949; 17,951	17,970	
2016	Soft wheat	15	17,973; 17,975; 17,978; 17,979; 17,983; 17,984; 17,987; 17,989	17,982	17,977; 17,981	17,974; 17,976, 17,980; 17,988	
	Durum wheat	3	17,985; 17,986		17,990		
	Total	134	70	11	24	26	3
